# The Conundrum of XenomiRs and Human Health

**DOI:** 10.1016/j.advnut.2025.100510

**Published:** 2025-09-11

**Authors:** Federica Tambaro, Carmen Gallicchio, Simona Orlando, Sara Carnevale, Maurizio Muscaritoli

**Affiliations:** 1 Department of Translational and Precision Medicine, Sapienza University of Rome, Rome, Italy; 2 Clinical Nutrition Unit, Ospedale Belcolle, Viterbo, Italy

**Keywords:** xenomiRs, cross-kingdom regulation, nutrimiRomics, diet, bioactive compounds, microRNA, plant-based food, personalized nutrition, precision medicine, public health

## Abstract

MicroRNAs (miRNAs) are small, noncoding RNAs involved in posttranscriptional gene regulation in both animal and plant. miRNAs derived from edible plants, referred to as xenomiRs, are proposed to cross-kingdom barriers and to modulate mammalian gene expression. However, this dietary xenomiR hypothesis remains controversial, given numerous inconsistencies and conflicting evidence regarding stability, bioavailability, and functionality of xenomiRs in mammals. Despite promising findings, including reports of plant-derived miRNAs influencing mammalian gene expression in vitro and in animal models, evidence remains inconclusive in humans. Several independent investigations have reported contradictory findings, emphasizing reproducibility lack in identifying and validating the transfer of xenomiRs from plants to mammals, which has raised concerns about the robustness, reliability, and biological significance of some results. Additionally, no direct molecular evidence currently demonstrates that plant xenomiRs bind to mammalian silencing machinery. Although the concept of plant-derived xenomiRs holds significant potential, future research must address unresolved technical and biological limitations. If validated, this hypothesis may represent a novel avenue for epigenetic modulation through dietary intervention in precision medicine and personalized nutrition. This comprehensive narrative review critically provides an overview of the dietary xenomiR hypothesis, specifically focusing on those derived from edible plants. We summarize the current evidence regarding xenomiR cross-kingdom communication potential, and we discuss technical and biological challenges that impede their validation. We also explore the speculative yet plausible scenario that dietary miRNAs may act locally on the microbiota rather than systemically.

**Statements of Significance**

This review provides a timely and critical synthesis of the dietary xenomiR hypothesis, addressing supporting data and interpretations related to cross-kingdom communication, the ongoing controversy surrounding the phenomenon, and the lack of mechanistic insight into how these exogenous miRNAs might influence human health and disease. It highlights unresolved methodological challenges and offers clear directions for future mechanistic research to advance this controversial field.

## Introduction

A well-balanced diet supports physiological homeostasis and may contribute to disease prevention not only through essential nutrients but also through bioactive molecules found in plant-based foods [[1], [2], [3], [4]]. Studies have pointed to a clear association between the beneficial effects of dietary products and the modulation of specific endogenous microRNAs (miRNAs) [5], small noncoding RNA molecules involved in regulating critical biological processes [6].

A novel dimension has recently emerged, sparking growing interest into an intriguing, yet unresolved question: do the beneficial effects of certain foods stem solely from their nutraceutical components influencing endogenous miRNAs, or could they also be attributed to the miRNAs naturally present within these foods? This has given rise to the concept of cross-kingdom communication, most notably illustrated by the hypothesis that exogenous food-derived miRNAs (mainly derived from edible plants) collectively referred to as xenomiRs, can interact with mammalian gene regulatory networks, potentially influencing health and disease [7,8]. This hypothesis, if validated, could redefine our understanding of exogenous plant-derived miRNA in foods as a functional component of nutrition, opening new avenues in epigenetic modulation, nutrigenomics, and precision medicine. However, although plausible, our current understanding is limited to speculative studies, as no conclusive molecular-level evidence confirms the mechanisms by which xenomiRs derived from edible plants could potentially impact mammalian gene expression [9]. A growing body of evidence has revealed numerous inconsistencies and conflicting results concerning these plant miRNAs' stability, bioavailability, and functionality in mammalian systems, particularly in humans. These discrepancies have raised important questions about technical and biological limitations that hinder reproducibility and mechanistic clarity.

In this comprehensive narrative review, we provide a critical overview of the dietary xenomiR hypothesis, with a particular focus on miRNAs derived from edible plant sources. Unlike other reviews published on this topic, the present one holds the novelty of summarizing the current experimental evidence supporting and challenging the idea of cross-kingdom communication, exploring the key methodological and conceptual hurdles, and discussing unresolved questions regarding the stability, absorption, transport, and biological relevance of plant-derived miRNAs in mammalian systems. Rather than attempting to confirm or dismiss the hypothesis of xenomiR-driven gene silencing in mammalian cells, our goal is to clarify the state of the science by highlighting gaps in knowledge that currently hinder its validation. We further aim to refocus the discussion on other mechanistically plausible targets, and to outline strategic directions for future research to test the validity and therapeutic potential of dietary xenomiRs.

## Exploring the miRNA World: Insights into Animal and Plant Differences

miRNAs are a class of small noncoding RNAs, typically 19–25 nucleotides in length, that are highly conserved among species [10]. Because their discovery in 1993 [11,12], miRNAs have been shown to play essential roles in gene regulation and numerous biological processes at both the intracellular and intercellular levels [13]. Aberrant miRNA expression has been associated with a wide range of diseases, including cancer, neurodegenerative, inflammatory, and cardiovascular diseases [14]. Along this line, miRNAs have gained increasing clinical attention and progressively emerged as powerful tools in diagnostics and therapeutics [15]. Their attractive role as disease biomarkers and therapeutic targets is increasingly being explored in precision medicine [16].

**FIGURE 1:**
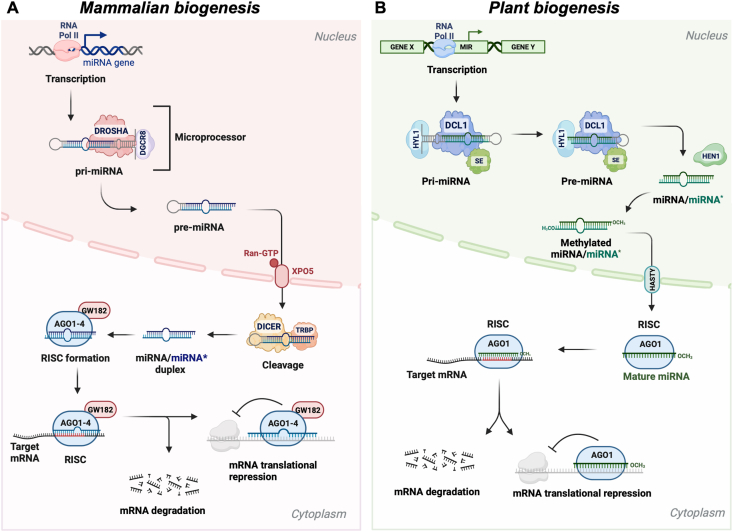
Canonical biogenesis of microRNAs (miRNAs) in animals and plants. (A) In mammals, miRNAs are predominantly transcribed by RNA polymerase II (Pol II) as long primary transcripts (pri-miRNAs) with characteristic stem-loop structures. The microprocessor complex, which includes the RNase III-type endonuclease Drosha and its essential cofactor DiGeorge syndrome critical region 8 (DGCR8), mediates the first nuclear cleavage, producing a ∼70-nucleotide precursor miRNA (pre-miRNA) with a 2-nucleotide 3′ overhang. The pre-miRNA is then actively exported to the cytoplasm by Exportin-5 in association with Ras-related nuclear protein/GTP-binding nuclear protein RAN (Ran-GTP). Once in the cytoplasm, pre-miRNA is further cleaved by the RNase III-type endonuclease Dicer, in association with RNA-binding proteins, such as transactivation response element RNA-binding protein (TRBP), yielding an imperfectly matched miRNA duplex of ∼18–25 nucleotides in length. The guide strand is incorporated into the RNA-induced silencing complex (RISC), composed of argonaute (AGO) proteins and other cofactors such as members of the GW182 protein family (TNRC6A, TNRC6B, and TNRC6C). The guide miRNA mediates posttranscriptional gene silencing via mRNA degradation or translational repression. (B) In plants, miRNAs are primarily transcribed by RNA Pol II as pri-miRNAs. Plant miRNA biogenesis occurs entirely in the nucleus. The RNase III enzyme dicer-like 1 (DCL1), along with cofactors hyponastic leaves 1 (HYL1) and serrate (SE), processes pri-miRNAs into pre-miRNAs and subsequently into mature miRNA duplexes through sequential cleavages. These duplexes are 2’-O-methylated at their 3′ ends by the methyltransferase Hua enhancer-1 (HEN1), which protects them from degradation. The mature duplex is exported to the cytoplasm via HASTY. The guide strand is incorporated into the RISC complex, predominantly stabilized by AGO1, to mediate gene silencing via either mRNA cleavage or translational inhibition.

As reported in the primary public repository, thousands of miRNAs have been identified in animals and plants [17]. Although animal- and plant-derived miRNAs share fundamental features, there are substantial differences in their functions, genomic locations, biogenesis pathways, and target recognition [18]. In animals, miRNAs regulate key cellular functions, such as proliferation, differentiation, apoptosis, metabolism, and immunity [19], whereas in plants, miRNAs control stress responses, homeostasis, metabolism, and growth [20]. Animal miRNAs typically originate from intragenic (or, less commonly, exonic) [[21], [22], [23]] or intergenic regions [24] and may be transcribed as clusters [25,26]. In contrast, plant miRNAs generally originate from intergenic regions or transposable elements [27] and are typically transcribed as single units [28]. Additionally, although both animal and plant miRNAs originate as primary transcripts (pri-miRNAs) transcribed by RNA polymerase II [29], they follow distinct biogenesis routes, which are summarized in Figure 1 and briefly described below.

In animals, pri-miRNAs are processed by the microprocessor complex (Drosha and DiGeorge syndrome critical region 8) [30,31] into precursor miRNAs (pre-miRNAs) [32], and exported to the cytoplasm via Exportin5/Ras-related nuclear protein/GTP-binding nuclear protein RAN [33,34], where Dicer complexed with RNA-binding protein (transactivation response element RNA-binding protein) [35], cleaves them into miRNA duplexes of ∼18–25 nucleotides in length. The guide strand binds RNA-binding proteins [argonaute (AGO), proteins, and members of the GW182 protein family] to form the RNA-induced silencing complex (RISC), which mediates gene silencing through translational repression or mRNA decay [36,37]. In contrast, plant miRNA biogenesis occurs entirely in the nucleus, with pri-miRNAs processed by dicer-like 1 [38], in association with hyponastic leaves 1 [39], and serrate [40], to generate both the pre-miRNA and the mature miRNA duplex [41]. Most mature plant miRNA duplexes are methylated at their 2’-OH of the 3’-end by Hua enhancer-1 [42], protecting them from degradation [43]. In addition, a specific GC-rich sequence signature has been implicated in ensuring accurate processing during biogenesis [44]. Mature plant miRNAs are exported to the cytoplasm by HASTY, the plant homolog of Exportin5 [45], selectively loaded into RISC, likely stabilized by AGO1 [46], to mediate gene silencing [13], through mRNA cleavage or translational repression [47].

Animal miRNAs often bind imperfectly to multiple targets [48], primarily within 3’-UTRs (untranslated regions) [49], enabling broad and complex regulatory networks [50]. In contrast, plant miRNAs typically exhibit near-perfect base-pairing complementarity to their targets [28], primarily binding within the coding regions or UTRs [51], and usually recognize a single gene due to this high degree of complementarity [28].

## Food and miRNAs: From Endogenous Modulation to Cross-Kingdom Communication

**FIGURE 2:**
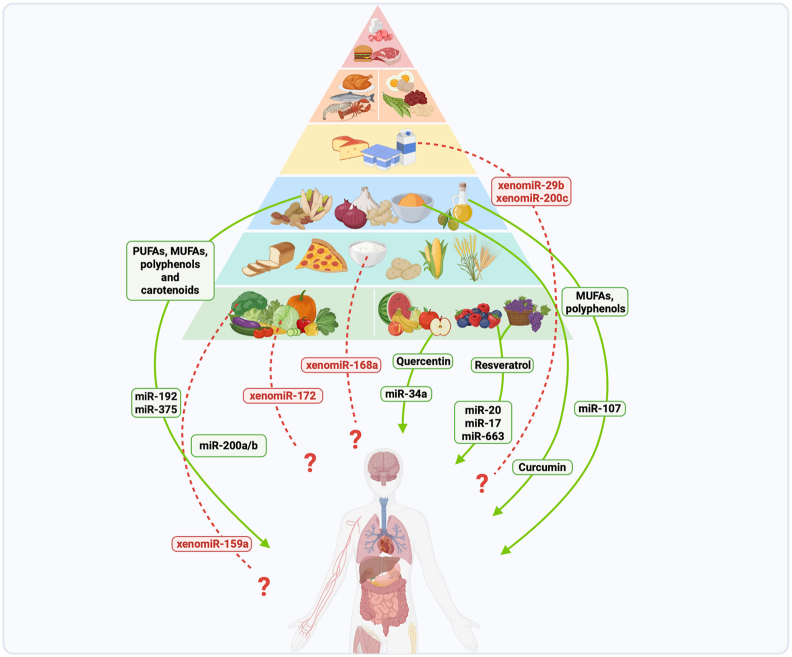
Schematic overview of dietary regulation of endogenous miRNA and xenomiR. This figure illustrates the emerging complexity of dietary miRNA interactions, highlighting both the well-established nutrigenomic regulation of endogenous microRNAs (miRNAs) and the still speculative yet compelling hypothesis that dietary xenomiRs may act as cross-kingdom regulators of gene expression. It depicts the Mediterranean diet pyramid, emphasizing various food groups and their bioactive components, such as PUFAs, MUFAs, polyphenols, and carotenoids, which have been shown to influence endogenous miRNA expression (indicated by solid green arrows). Examples include curcumin, quercetin, and resveratrol, which influence specific endogenous miRNAs implicated in various pathways. Red dashed arrows and question marks represent the possible but unconfirmed impact of food-derived exogenous miRNAs (xenomiRs) on human health, highlighting the ongoing uncertainty surrounding their systemic uptake and regulatory functionality.

Diet has long been recognized as a key player in regulating metabolic processes, influencing human health and disease [1]. For instance, Mediterranean diet, characterized by high consumption of fruits, vegetables, whole grains, and healthy fats, has been extensively studied for its potential beneficial effects [52]. These effects are partly mediated through the modulation of endogenous miRNA expression by bioactive dietary components [53] (Figure 2). Notably, extra virgin olive oil and pistachios have been shown to influence glucose and lipid metabolism via modulation of miR-107, miR-192, and miR-375 [54]. Polyphenols such as resveratrol have also been shown to regulate miRNAs involved in mitochondrial function and tumor suppression [55,56]. Other compounds, such as quercetin and curcumin, also modulate miRNAs involved in inflammation and apoptosis [57,58].

The well-established interaction between diet and endogenous miRNAs lays the groundwork for the emerging field of NutrimiRomics, which integrates omics sciences into nutritional research to explore how diet influences gene expression [[59], [60], [61]]. However, the specific mechanisms through which diet enhances endogenous miRNA expression are still to be fully determined. A key emerging aspect of NutrimiRomics is the dietary xenomiR hypothesis, which suggests that food-derived miRNAs may regulate host gene expression, with potential implications for human health and diseases [59]. This hypothesis is supported by detecting endogenous miRNAs in body fluids [62], often attributed to their encapsulation within extracellular vesicles (EVs) [63], lipid bilayer-bound particles secreted by nearly all eukaryotic cells that transport nucleic acids, proteins, and metabolites [64]. Mammalian EVs are classified as exosomes, microvesicles, and apoptotic bodies [65], whereas plant-derived EVs share similar features to mammalian exosomes [66]. EVs are widely recognized for their role in intra- and intercellular communication [67], although their exact functions in the intercellular spread of miRNAs remain poorly understood [67,68]. However, the functional impact of dietary xenomiRs on humans, whether or not they are shielded within EVs, remains debated. The possibility that these food-derived miRNAs can exert biological effects in the host is compelling, and the exact mechanisms through which they might do so have yet to be consistently and unequivocally demonstrated. In the following sections, we will briefly summarize the evidence and controversies surrounding the ability of food-derived xenomiR on mammalian gene expression.

## Plant-Based xenomiRs

The presence of exogenous miRNAs in plasma was first highlighted in 2012 by Zhang et al. [69], who reported high levels of plant-derived miRNAs in human sera, including rice-derived miR-168a (*Oryza sativa*) that inhibited the LDL receptor adapter protein 1 (LDLRAP1) in mouse liver and increased plasma LDL levels after rice-feeding.

This groundbreaking research has prompted many scientists to investigate the intriguing properties of plant-derived xenomiRs (Table 1) [[69], [70], [71], [72], [73], [74], [75], [76], [77]]. Although beyond the aims of the present review, we also include a few examples of animal-derived xenomiRs, particularly those found in human and animal milk, to give a broader perspective on cross-kingdom communication (Table 2) [[78], [79], [80], [81], [82], [83], [84], [85]]. Liang et al. [71] found that miR-172 from *Brassica oleracea* persisted in multiple mouse tissues and feces ≤72 h postfeeding, demonstrating its resistance to gastrointestinal degradation. Chin et al. [72] showed that oral administration of synthetic miR-159a, abundant in broccoli, inhibited xenograft breast cancer growth in mice and reduced MYC protein levels by targeting transcription factor 7 in vitro. Similarly, treatment with miR-159a inhibited the proliferation of Caco-2 cells in a colorectal cancer model [73]. Link et al. [70] observed increased miR-168 in stool samples and gastrointestinal mucosa, but not in blood, of healthy subjects who followed a vegetarian diet for 5–7 d. MiR-156a, found abundantly in dietary green vegetables and rice, reduced inflammation-induced adhesion in endothelial cells by targeting junctional adhesion molecule A in vitro [74]. More recently, the effects of aqueous *Moringa Oleifera* seed extract or its purified miRNAs decreased intracellular lipid accumulation and prevented metabolic dysregulation in both animals and in vitro obesity models [86]. In one of the few human studies on dietary xenomiRs, serum levels of miR-2911 were found elevated in 3 healthy volunteers after consuming 30 g/d of *Lonicera japonica* (honeysuckle), a Chinese herb that is used to treat influenza infections [75]. This study was later extended to patients with SARS-CoV-2 infection and linked to antiviral activity against influenza A viruses [87], and varicella-zoster virus [76].

**TABLE 1:** Overview of reported plant-derived xenomiRs.

xenomiR	Plant source	Study context	Key findings	References
miR-168a	Rice (*Oryza sativa*)	Human sera, in vitro, animal models	Inhibition of LDLRAP1 expression	[69,70]
miR-172	Cabbage *(Brassica oleracea)*	In vitro and animal models	Ability to cross the GI tract, to enter the bloodstream, and to reach different organs	[71]
miR-159a	Broccoli, soybeans	Human sera, in vitro, animal models	Decrease in MYC protein levels by targeting TCF7; inhibition of neoplastic cell proliferation; inhibition of colorectal cancer cell proliferation	[72,73]
miR-156a	Green vegetables and rice	In vitro	Decrease of inflammation-induced cell adhesion by targeting JAM-A	[74]
miR-2911	Honeysuckle (*Lonicera japonica*)	Human sera, in vitro*,* animal models	Inhibiting viral replication	[[75], [76], [77]]

Abbreviations: GI tract, gastrointestinal tract; JAM-A, junctional adhesion molecule A; LDLRAP1, LDL receptor adapter protein 1; miR, microRNA; MYC, myelocytomatosis oncogene; TCF7, transcription factor 7.

**TABLE 2:** Overview of reported xenomiRs from animal sources.

xenomiR	Source	Study context	Key findings	References
miR-29b, -39, -166a, -200c	Milk	Human sera, in vitro, animal models	Absorption and resistance in the digestive processes during gastrointestinal transit	[[78], [79], [80], [81]]
miR-1, -99a, 133a-3p, -154, and -206	Meat	In vitro, animal models	Implicated in the modulation of genes involved in lipid and glucose metabolism	[82,83]
miR-142, -181b, and miR-30a	Chicken-egg	Human sera, in vitro, animal models	Egg-miRNA exosome may regulate spatial learning and memory function	[84,85]

Abbreviation: miR, microRNA.

## Conflicting Evidence on Food-Derived Plant xenomiRs and Current Challenges

Despite early enthusiasm, evidence of xenomiRs being acquired through dietary sources and their relevant biological effects remains highly inconsistent and controversial.

Dickinson et al. [88] found no increase in miR-168a levels or downregulation of LDLRAP1 in humans or mice fed with plant-rich diets, attributing Zhang’s early findings to nutritional imbalance rather than a cross-kingdom effect. Tosar et al. [89] and Snow et al. [90] further argued that Zhang’s results may stem from cross-contamination during sequencing processes or technical artifacts, with Snow unambiguously showing no detectable xenomiRs in the plasma of healthy humans and mice fed a diet enriched in certain plants. Witwer et al. [91,92], although able to detect a low amount of plant-derived xenomiRs in the plasma of *Macaca nemestrina*, attributed these results to nonspecific amplification, reaffirmed in a 2018 study that artifact contamination was the most likely explanation for xenomiRs detection. Similarly, Huang et al. [93] found no significant differences in serum or tissues from mice fed a plant-based diet. Fromm et al. [94] and Tosar et al. [89] reported that transcriptomic and bioinformatic contamination might explain the reasons for xenomiRs’ conflicting evidence. Reanalyzing Baier’s study [78], with a custom OpenArray, Auerbach et al. [95] found no significant altered miRNA signals postmilk ingestion, indicating that dietary xenomiRs do not transfer into human circulation. In a large-scale analysis of 824 human datasets, Kang et al. [96] concluded that xenomiRs likely originate from technical artifacts rather than dietary intake due to their extremely low abundance.

The inconsistencies mentioned above are primarily attributed to the technical limitations related to the methodologies used to identify the presence of plant-derived xenomiRs in mammalian body fluids and tissues. Commonly cited issues include differences in experimental design flaws, background noise in RT-qPCR, cross-contamination during library preparation and sequencing (e.g., poor primer design, nonspecific assay), and inefficient bioinformatics pipelines [89,94,97,98]. Furthermore, sample collection procedures [99], and RNA purification methods [98], have also been shown to influence detection outcomes, further complicating data interpretation.

These methodological inconsistencies represent a significant source of controversy, as they compromise reproducibility, hinder direct comparisons across studies, and ultimately weaken the reliability and credibility of the conclusions drawn, thereby delaying scientific progress. Thus, these multiple technical challenges must be consistently and unequivocally addressed before the hypothesis of cross-kingdom delivery of exogenous plant-based miRNA via food ingestion can be definitely accepted.

In the following sections, we will explore current gaps in knowledge and inconsistencies concerning the stability and bioavailability of plant-derived xenomiRs, the mechanisms underlying their transport from the intestinal lumen into the blood and ultimately into target cells, and the minimum effective concentrations needed to elicit functional responses. These aspects are critical to clarify the current distance between existing evidence and the full validation of dietary plant xenomiRs cross-kingdom communication and their functional relevance in human health and disease.

## Stability and Bioavailability of Plant xenomiRs

A key challenge in understanding cross-kingdom communication lies in determining the ability of plant-derived xenomiRs to survive food processing and to remain bioavailable postingestion. High-temperature processing (e.g., cooking or boiling, which is sometimes necessary) can lead to miRNA degradation [100,101]. Moreover, to exert biological effects in humans, plant-derived xenomiRs must also tolerate and survive the harsh conditions of the oral cavity (e.g., RNases and lytic enzymes) and the acidic/basic environment of the gastrointestinal tract (GIT) [102]. However, our current understanding of how xenomiRs overcome these obstacles remains limited, largely speculative, and fragmented. Many studies in favor of the cross-kingdom communication hypothesis rely heavily on the premise that EVs are the primary protective carriers of plant xenomiRs. The presence of circulating endogenous miRNAs in human body fluids [64], is frequently cited to support this view. Additionally, the identification of plant EV-rich miRNAs in 11 fruits and vegetables [103], as well as the detection of some plant-derived miRNAs in milk-derived EVs [79], along with the demonstration that some plant-derived miRNAs could remain stable during food processing and cooking [104,105], has reinforced the hypothesis. This assumption is further supported by the demonstrated advantages offered by plant-derived EVs, including accessibility, biosafety, GIT resistance, and immune tolerance [106], along with their proven stability and biocompatibility as natural drug carriers and oral delivery vehicles for RNA-based therapies [77,107]. Notably, although dairy-derived xenomiRs were significantly reduced during pasteurization and homogenization [108], certain plant-derived xenomiRs appeared more resistant to degradation during cooking and digestion, potentially reaching the distal segments of the GIT [109]. On the basis of the understanding that functional extracellular miRNAs require protection [110], many studies suggest that plant-derived xenomiRs maintain higher stability due to their packaging into EVs, allowing them to evade degradation by digestive enzymes [110,111]. Although in vitro experiments have shown that broccoli-derived EVs can withstand simulated GIT and be internalized by intestinal epithelial cells (Caco-2), and a separate bioinformatic study has proposed resistance of plant miRNAs to food processing and GI digestion [[112], [113], [114]], these results are limited to in vitro and in silico models and remain to be validated in vivo*.* Moreover, although it is reasonable to assume that both EV packaging is sufficient to ensure plant xenomiR stability and bioavailability, substantial uncertainties still persist. Some studies have detected plant-derived xenomiRs in human fluids and tissues [115], others in feces but not in blood [70], whereas several have failed to detect them in any animal or human body fluids [[116], [117], [118]]. These observations highlight that EV-associated xenomiRs may not be fully protected from degradation, suggesting that the concept of complete resistance is likely oversimplified and may be more accurately described as partial resistance.

Furthermore, the prevailing reliance on EVs as the exclusive protective mechanism for plant-derived xenomiRs may limit our current understanding. Although EVs remain a compelling vehicle, the assumption that they are the only functional carriers of plant-derived xenomiRs may be an oversimplification. Indeed, plant-derived xenomiRs may also be stabilized by alternative structures, such as ribonucleoprotein complexes [119], lipid-based carriers [120], or packaging into apoptotic bodies [121], all of which may contribute to their enhanced stability and bioavailability, independently of EV packaging. However, their role in protecting dietary plant miRNAs against gastrointestinal degradation remains largely unexplored.

To overcome these issues, it is essential to refine isolation techniques of plant-derived EV, particularly in light of the complex structure and heterogeneity of plant cells [122], taking into account other xenomiRs’ protective mechanisms, as well as the structural differences between plant- and animal-derived miRNAs. Indeed, the enhanced stability of plant-derived xenomiRs has also been largely attributed to their 3′-end 2′-O-methylation [123] and to their highly organized stem-loop secondary structures [124]. A recent study on endogenous miR-21-5p in human nonsmall cell lung cancer showed that 3′-terminal 2′-O-methylation can enhance miRNA resistance to exonuclease degradation and increase AGO2 loading efficiency [125]. However, this evidence comes from a tumor model and refers to an endogenous mammalian miRNA, and thus should be interpreted with caution when extrapolated to dietary plant miRNAs. Notably, atypical sRNAs such as MIR2911 have shown exceptional digestive stability [126], likely due to specific sequence and structural features rather than canonical miRNA characteristics [127]. However, these findings are limited to a single noncanonical molecule and do not generalize to the broader class of dietary plant miRNAs. As such, these results reinforce the need for critical evaluation of each specific case.

Despite the multiple mechanisms proposed and discussed above, no definitive mechanism has yet been established to explain the stability of plant-derived miRNAs and their resistance to digestion and processing.

## Barriers to Uptake: From Digestion to Integration

Further complicating the hypothesis of xenomiR-mediated cross-kingdom communication is the challenge of identifying if and how these molecules are absorbed. An additional uncertainty lies in pinpointing the specific region of the GIT where this absorption might take place, making it essential to determine whether xenomiRs act in proximal or distal segments of the GIT. Specifically, if xenomiRs do reach the distal gut region, it remains unclear whether and how they interact with microbiota and immune cells, and if and how they cross the epithelial layer to enter the systemic circulation. The gut barrier is a multilayer structure composed of intestinal epithelial cells that form a continuous physical barrier, coated by a sticky, viscous layer of mucus, which impedes the uptake of potentially harmful environmental particles [128]. In addition, the colon and small intestine are largely populated by gut microbiota and immune cells, which produce a wide range of enzymes that can contribute to miRNA degradation [128]. The proposed mechanisms allowing intestinal content to cross the GIT epithelium include transcellular transport (through cells via transcytosis or protein transporters), paracellular pathways (between cells via EVs facilitation) [129], or immune cell sampling (e.g., by dendritic cells) [130]. However, none of these routes has been definitely shown to be involved in xenomiR intestinal uptake.

## Cross-Kingdom Gene Regulation: How Dietary xenomiRs Functionally Integrate into Mammals

Studies of the miRNA regulatory network have demonstrated shared biological pathways between animals and plants, particularly in posttranscriptional gene regulation [17,131,132]. However, a major gap in current knowledge is whether the assumption of conserved posttranscriptional mechanisms between plant and animal miRNAs is sufficient to support the hypothesis of cross-kingdom communication. The dietary xenomiR hypothesis proposes that exogenous miRNAs from edible plants may regulate endogenous genes in mammals by engaging the host RNA silencing machinery [97]. Although some studies have tested plant-derived miRNAs in vitro or in vivo by trying to assess their impact on mammalian targets, many implicitly assume that plant miRNAs operate via conserved mechanisms known for endogenous miRNAs in mammals, such as AGO binding and RISC incorporation, without directly validating these steps. This assumption, although widespread, has not yet been confirmed at the molecular level in higher eukaryotes and ultimately requires understanding of how xenomiRs are absorbed and internalized by host cells. This capacity has been largely inspired by studies on *Caenorhabditis elegans,* where extracellular double-stranded RNA (dsRNA) induced gene silencing by acting as templates for small interfering RNAs (siRNA) formation, either when added to the diet or expressed in bacteria consumed by the nematode [133,134]. In *C. elegans*, dsRNA uptake occurs via transmembrane channels called systemic RNA interference defective proteins 1 (SID-1) and SID-2 [135]. Although mammals do not express SID-1 or SID-2, they possess related paralogs (SID-T1 and SID-T2) [136], whose functions remain incompletely understood. However, the evidence for systemic RNA uptake in higher eukaryotes remains inconclusive. Some studies conducted in mice have suggested a possible role for SID-T1 in facilitating the uptake of dietary small RNAs such as MIR2911 [137,138]. However, this RNA species is not a canonical plant miRNA but a ribosomal RNA degradation fragment, and its classification as a functional miRNA has been questioned [92]. Therefore, the translational relevance of these findings in humans remains limited and controversial.

Importantly, unlike nematodes, dsRNA is not functionally processed in mammals, but is instead frequently recognized as a potential hazard, triggering innate immune responses through cellular sensors that detect foreign nucleic acids and initiate signaling pathways to suppress pathogen replication [139,140]. This could also result in the degradation of exogenous miRNAs. Therefore, one should exclude the possibility that xenomiRs reach mammalian systems in the form of duplexes. Instead, they are likely to be delivered in a protected form, either complexed with their native plant AGO or protected in their EVs. Assuming that these protective carriers enable xenomiRs to successfully enter mammalian cells through an unknown transport mechanism or through EV-membrane fusion, a major challenge lies in elucidating the mechanisms through which xenomiRs may integrate into host regulatory systems. For any functional outcome, these miRNAs must be released from plant-derived carriers and successfully interact with endogenous RISC components [141]. However, AGO-miRNA complexes are highly stable [142,143], and so far, no molecular mechanism has been described for AGO-mediated small RNA transfer. Although computational analyses predict potential plant-derived miRNA binding sites in mammalian genomes, these interactions have not been conclusively validated, and no direct evidence has shown plant miRNAs binding to mammalian AGO proteins [144]. Moreover, due to differences in sequence and secondary structure, it would not be possible for endogenous AGO to recognize exogenous miRNAs [145], raising concerns about relying solely on computational predictions, which cannot substantiate claims of cross-kingdom regulation.

## Required Intake of Plant-Derived xenomiRs for Effective Cross-Kingdom Gene Regulation

Although the concept of cross-kingdom regulation by plant-derived xenomiRs is intriguing, current evidence suggests that vegetables do not supply sufficient xenomiRs to achieve biologically relevant effects in mammals, nor do they support their functional significance within the host. Indeed, a major challenge lies in the limited information available about the minimum concentration of plant xenomiRs required to exert regulatory effects in mammals. For miRNAs to exert measurable biological effects, their intracellular concentration must be sufficient to compete for binding with target transcripts. Although theoretical models propose that even minimal concentrations might influence gene expression [[146], [147], [148]], estimates suggest that biologically active miRNA concentration should range from 100 to 10,000 copies per cell [149]. However, dietary plant xenomiRs, when detectable *in vivo*, often appear at levels several orders of magnitude below this threshold. Estimates suggest that an average adult human harbors around 40 trillion (4 × 10^13^) cells [150], and delivering just 1000 copies of a plant-derived miRNA to each cell would require consuming tens to thousands of kilograms of miRNA-rich foods. For instance, to be functional, ∼67 kg of cooked rice would be required for miR-168a [151], or 1670 kg of cantaloupe for miR156a [90]. This implies that the daily intake of plant-derived xenomiRs required for functional effects would necessitate unrealistically high food consumption. Moreover, plant miRNA content varies greatly across species [152], and exhibits a tissue-specific pattern strongly influenced by abiotic stressors [153]. At such thresholds, the low abundance of xenomiRs found in plant food is unlikely to be sufficient to exert biological effects on the host organism.

## Plant-Derived xenomiRs and Human Health: A Yet Unfulfilled Promise

The potential of plant-derived xenomiRs to modulate gene expression in mammals holds promise for translating these findings into therapeutic or nutritional strategies, including the modulation of disease susceptibility through epigenetic modifications and the potential to improve clinical outcomes. If properly validated, the ability of specific foods or dietary patterns to modulate gene expression through plant-derived miRNAs may open novel opportunities for targeted dietary interventions. This could be particularly relevant in clinical settings such as chronic diseases and cancer, where body composition changes, which often reduce responsiveness to treatments and increase patient mortality [154], are associated with differential modulations of endogenous miRNAs [[155], [156], [157]]. Understanding whether and how food-borne miRNAs influence host gene expression might support the development of miRNA-enriched dietary supplements or functional foods tailored to improve patient outcomes in precision nutrition.

Although animal and in vitro studies provide some evidence of xenomiR uptake and functionality, the findings, particularly in humans, are still controversial and inconsistent, leaving this hypothesis largely inconclusive (Table 3). The discrepancies are often attributed to differences in methodology, such as design, detection, and interpretation. Our aim is not to confirm or dismiss the hypothesis of xenomiR-driven gene silencing in mammalian cells, but to offer a biologically plausible reinterpretation of the available evidence. Critically, the biological mechanisms underpinning plant-derived xenomiR stability, bioavailability, and systemic transport remain poorly defined. The quantities of dietary xenomiRs required to elicit systemic regulatory effects appear unrealistically high, exceeding plausible dietary intake levels. This further challenges the notion of plant xenomiRs to achieve intracellular concentrations sufficient to impact host gene expression. Additionally, beyond computational predictions of plant xenomiR-mammalian mRNA interactions, the absence of confirmed molecular mechanisms for both cellular uptake and functional integration into host regulatory systems in higher eukaryotes leaves the cross-kingdom regulation hypothesis largely speculative.

**TABLE 3:** The conundrum of xenomiRs and human health: summary of the current issues and study limitations.

Open questions	Available evidences	Future research directions
Can plant miRNAs resist food processing and digestion?	Limited in vitro stability observed for some EV-encapsulated or atypical small RNAs; strong degradation under physiological conditions	Standardized digestion protocols; in vivo models to track degradation and resistance
Can xenomiRs cross the gastrointestinal barrier?	Some in vitro studies suggest apical uptake by intestinal cells; in vivo translocation and systemic availability remain unconfirmed	Improved models of intestinal permeability (e.g., coculture systems, organoids); in vivo absorption studies using traceable dietary miRNAs
Which factors confers xenomiRs stability?	Preliminary evidence suggests 2′-O-methylation, stem-loop secondary structures, and EV encapsulation may enhance resistance	Systematic mapping of chemical modifications; correlation with resistance; exploration of additional protective mechanisms (e.g., protein binding, plant-derived lipid vesicles)
How are plant miRNAs transported systemically?	No consistent detection in human circulation; low abundance and degradation issues	Sensitive RNA detection; use of labeled synthetic miRNAs; plasma exosome profiling
Do plant miRNAs interact with mammalian RISC complexes?	No direct evidence of AGO loading for canonical plant miRNAs; functional association remains speculative due to species-specific incompatibility and absence of structural validation	Cross-species AGO binding assays; AGO immunoprecipitation followed by sequencing; structural modeling and crystallographic studies
Are dietary miRNAs functionally active in host gene silencing?	Reported effects are mostly based on in vitro systems or in silico target predictions; many lack validation, dose–response, or mechanistic plausibility	Integration of bioinformatics with experimental pipelines to control for artifacts; functional validation of predicted targets; improved design of in vitro and in vivo models
Are dietary miRNAs bioavailable in humans?	Data are inconsistent and mostly limited to animal or in vitro studies; detection in humans often near threshold and method-dependent	Human intervention studies with validated detection pipelines; time-course analysis to assess miRNA dynamics and clearance
What doses of plant miRNAs are required for functional effects?	Available data suggest that endogenous levels of plant miRNAs in foods are extremely low and often insufficient to elicit regulatory activity	Comprehensive quantification of miRNAs in food before and after processing; correlation with biological activity thresholds; dose–response assessments in controlled models

Abbreviations: AGO, argonaute; EV, extracellular vesicles; miRNAs, microRNAs.

Until these critical gaps are thoroughly addressed, the promise of edible plant-derived xenomiRs mediating cross-kingdom communication remains disappointingly elusive.

A more plausible scenario may be that a small fraction of dietary plant-derived xenomiRs that survive digestion barriers exerts localized effects on the gut microbiota [158], rather than exerting systemic or distal effects on the host. Although mechanistic validation is still lacking, the presence of abundant RNases and microbial nucleases in the GIT [159] may facilitate the breakdown of xenomiRs into nucleotide fragments that can be beneficially used by the microbiota. This suggestion is grounded in the mechanistic constraints outlined above: in the absence of validated uptake and systemic distribution, the GIT and the gut lumen remain a plausible site where small amounts of plant-derived xenomiRs, having survived the sequential degradative processes of the upper digestive tract (i.e., oral cavity, stomach, small intestine), may persist and interact with microbial communities.

Indeed, the possibility that plant-derived xenomiRs might exert direct intraluminal effects should not be excluded. Notably, evidence exists that diet can influence the modulation of the gut microbiota [160], and that endogenous miRNAs modulate bacterial homeostasis by shaping the microbiome profile and promoting the growth of commensal and probiotic bacteria [161,162]. In a similar manner, plant-derived xenomiRs might interact with the gut microbiome, potentially supporting the proliferation of beneficial bacteria [[163], [164], [165]]. Although no direct mechanisms have yet been demonstrated, and although the body of evidence is still limited, including uncertainty regarding whether their absorption take place in the proximal or distal segments of the GIT, one should not exclude that the potential for microbial sensing or utilization of exogenous RNAs remains biologically compelling. Such interactions might involve the contribution of nucleotide fragments to microbial balance [166]*.* Therefore, although we acknowledge that this is a speculative model based on preliminary data, we propose it as a testable hypothesis rather than a conclusion.

In this context, regardless of their ability to be absorbed or taken up by the GIT, degraded xenomiRs may act as nucleotide donors, supporting microbial metabolism and contributing to host–microbe cross-talk. This redirection of focus, from systemic gene silencing to local microbiome-mediated effects, may represent a more biologically grounded and experimentally accessible hypothesis.

In conclusion, at the core of this scientific debate lies the central conundrum of how, and to what extent, xenomiRs influence human health and disease. However, before definitively accepting or rejecting the hypothesis of plant-derived xenomiRs as mediators of cross-kingdom communication, it is essential to address critical gaps by providing clear mechanistic models and establishing consensus-driven methodological guidelines to clarify their biological relevance and potential functionality. One should also consider that, although the systemic uptake and intracellular activity of dietary plant xenomiRs remain unproven, their potential role in shaping the gut microbiome warrants deeper investigation. If confirmed, this alternative route could complement or replace the hypothesis of direct xenomiR-host transcript interaction, paving the way to a better understanding of the novel role of the gut microbiota as a pivotal system in cross-kingdom communication.

Addressing these research gaps is not only essential to evaluate their true biological relevance and translational potential, but also of potential public health relevance. If future studies demonstrate that plant-derived xenomiRs can survive digestion, be absorbed, and exert regulatory functions in humans, this could lay the groundwork for defining dietary recommendations based on miRNA content. Foods or dietary patterns might be selected, enriched, or fortified to deliver functional levels of miRNAs, particularly in populations with therapeutic requirements. This could lead to the formulation of novel dietary supplements or functional foods aimed at supporting gene regulation, immune function, or metabolic balance.

Clearly, as discussed throughout this review, many questions must be answered before xenomiR-based interventions can be realistically implemented.

These steps appear to be essential for validating or dismissing this controversial hypothesis and ensuring meaningful scientific progress.

## Author contributions

The authors’ responsibilities were as follows – FT, MM: revised and edited the final content; and all authors: conceptualization, wrote sections of the review and read and agreed to the published version of the manuscript.

## Funding

The author(s) acknowledge the support of grant PE00000003 (decree 1550, October 11, 2022) ON Foods—Research and innovation network on food and nutrition Sustainability, Safety and Security—Working ON Foods) from the Italian Ministry of University and Research (CUP D93C22000890001) under the National Recovery and Resilience Plan (NRRP), funded by the European Union—NextGenerationEU.

## Conflict of interest

The authors declare that they have no known competing financial interests or personal relationships that could have appeared to influence the work reported in this article.
